# Incidence of Posterior Capsule Rupture During Phacoemulsification Cataract Surgery Among Patients Treated With Intravitreal Bevacizumab Injections

**DOI:** 10.7759/cureus.70297

**Published:** 2024-09-26

**Authors:** Duaa T Daradkeh, Fedaa A Smadi, Hala K Haddad, Alaa A Smadi, Sura Y Habashneh, Zaineh A Shawareb

**Affiliations:** 1 Department of Ophthalmology, Royal Medical Services, Amman, JOR; 2 Department of Pharmacy, Royal Medical Services, Amman, JOR

**Keywords:** bevacizumab, diabetes mellitus, intravitreal, phacoemulsification, posterior capsular rupture

## Abstract

Objectives: To assess whether intravitreal bevacizumab injections increase the risk of posterior capsular rupture during phacoemulsification cataract surgery in diabetic patients.

Methods: A retrospective study that included diabetic patients who underwent phacoemulsification cataract surgery at King Hussein Medical Center of the Jordanian Royal Medical Services during a one-year period from January 2023 until January 2024. Patients were categorized into four groups. The first group did not receive an intravitreal bevacizumab injection; the second group received only one injection; the third group received two injections; and the fourth group received three or more injections. Results from the four groups were compared with a control group for patients who underwent phacoemulsification surgery and were not known to have diabetes mellitus and did not receive previous intravitreal injections. All injections were received within a period of one year before the time of phacoemulsification surgery. Inclusion criteria for the study groups included diabetic patients with a history of diabetes of more than 10 years with the presence of diabetic retinopathy. Exclusion criteria included patients who received intravitreal injections other than bevacizumab, previous intraocular surgery or trauma, and the presence of ocular pathology that led to an increased risk of posterior capsular rupture. The incidence of posterior capsular rupture was compared among these groups. A p-value was used to assess statistical significance and was considered to be statistically significant if ≤0.05.

Results: The number of patients in the control group (group 0) was 1440 patients with a mean age of 64.9, and in the study groups were 1306 patients. Group 1 included 244 patients (18.7%) with a mean age of 62.4 years, compared to 306 patients (23.4%) in group 2 with a mean age of 66.1 years, 352 patients (27%) in group 3 with a mean age of 64.2 years, and 404 patients (30.9%) in group 4 with a mean age of 63.1 years. Posterior capsular rupture occurred more significantly in patients with group 4. It occurred in 21 patients, representing 5.2% of them, compared to four patients (1.6%) of group 1, six patients (2%) of group 2, and eight patients (2.3%) of group 3, while in the control group, the number of patients with posterior capsular rupture was 20 (1.4%).

Conclusion: Intravitreal bevacizumab in diabetic patients is considered a risk factor for posterior capsular rupture in phacoemulsification cataract surgery in patients receiving more than three injections.

## Introduction

Bevacizumab is a humanized monoclonal antibody medication against vascular endothelial growth factor (VEGF). It is used to treat several different cancers. Intravitreal bevacizumab is used off-label to treat several eye conditions, such as choroidal neovascularization in age-related macular degeneration, macular edema due to diabetes, or central retinal vein occlusion [1-3].

In addition to the occurrence of diabetic retinopathy in diabetic patients, those patients also develop cataracts and eventually will need phacoemulsification surgery [4]. It is one of the most common and successful surgical procedures nowadays that involves the emulsification of the lens nucleus using ultrasonic energy and aspiration of the emulsified material. Preservation of the intact lens capsule is imperative for the subsequent implantation of an intraocular lens. If posterior capsule rupture occurs, many complications can be encountered, such as vitreous prolapse, secondary glaucoma, retinal detachment, cystoid macular edema, and endophthalmitis [5].

Intravitreal bevacizumab injection has been implicated as a potential risk factor for posterior capsular rupture in cataract surgery [6-9]. The aim of our study is to assess whether intravitreal bevacizumab injections increase the risk of posterior capsular rupture during phacoemulsification cataract surgery in diabetic patients.

## Materials and methods

A retrospective study that included diabetic patients who underwent phacoemulsification cataract surgery at King Hussein Medical Center of the Jordanian Royal Medical Services during a one-year period from January 2023 until January 2024. File review of patients included the duration of diabetes from the medical records and the presence of diabetic retinopathy, which was confirmed by fluorescein angiography or optical coherent tomography. Data recorded also included a history of trauma and the presence of ocular pathology that may increase the risk of complications during surgery, such as small pupils, pseudoexfoliation, glaucoma, and uveitis. We excluded patients with such conditions as they may cause a risk of rupture of the posterior capsule. Also, we recorded whether the patient received a previous intravitreal injection one year before they underwent surgery and the number of injections given. The aim that we did not include injections that were received one year prior to surgery was to standardize data among patients in all groups.

Surgeries were done by senior ophthalmic surgeons. Pre-surgical assessment in our institution includes best-corrected visual acuity, slit lamp anterior segment examination, intraocular pressure measurement, fundus examination after mydriasis, and biometry using an intraocular lens master. Operative notes and complications were also analyzed. Surgeries were done under topical anesthesia with supplemental use of intracameral lidocaine. Phaco machine and settings during surgery were the same for all patients. A one-piece intraocular lens was inserted unless posterior capsular rupture occurred where a three-piece lens was inserted. Posterior capsular rupture during surgery can be identified by direct visualization, vitreous loss, difficulty of lens matter aspiration, drop of lens matter, sudden deepening or shallowing of the anterior chamber, and momentary pupillary dilatation. Patients are followed up one day after the surgery where local steroids and antibiotics are prescribed, then followed up one week later unless a complication occurs.

Our institution is a tertiary referral medical center. There is a specialized medical retina clinic where intravitreal injections are given. They are frequently used in our institution in treating proliferative diabetic retinopathy, macular edema, and age-related macular degeneration, and the long-term complications must be addressed. The technique of intravitreal injection is standard for all patients in our institution. Topical anesthesia is applied, followed by 5-10% Povidone-iodine drops and a sterile speculum. The injection is given using sterile conditions, wearing surgical gloves and a face mask with antibiotic instillation after the procedure. The site of the injection is 4 mm posterior to the limbus in phakic eyes. The dose of intravitreal bevacizumab used is 1.25 mg in 0.05 mL injected in a 27-gauge needle. Repeated injections are usually given one month apart, depending on the follow-up of patients by optical coherent tomography. Post-injection, patients are instructed not to rub their eyes in the first 24 hours, to avoid getting water in their eyes for a few days, to use antibiotic eye drops, and to be seen in a follow-up clinic one week post-injection unless patients feel pain or blurring of vision.

Results from the four groups were compared with a control group for patients who underwent phacoemulsification surgery and were not known to have diabetes mellitus and did not receive previous intravitreal injections. The first study group did not receive an intravitreal bevacizumab injection; the second group received only one injection; the third group received two injections; and the fourth group received three or more injections. All injections were received within a period of one year before the time of phacoemulsification surgery. Inclusion criteria in the study groups included diabetic patients with a history of diabetes of more than 10 years with the presence of diabetic retinopathy. Exclusion criteria included patients who received intravitreal injections other than bevacizumab, previous intraocular surgery or trauma, and the presence of ocular pathology that led to an increased risk of posterior capsular rupture. The incidence of posterior capsular rupture was compared among these groups. A p-value was used to assess statistical significance and was considered to be statistically significant if ≤0.05. Ethical committee approval of the Royal Medical Services was obtained with IRB number 35/122024.

## Results

The number of patients in the control group (group 0) was 1440 patients with a mean age of 64.9, and in the study groups were 1306 patients. The mean age was comparable in four groups: 62.4 years in group 1, 66.1 years in group 2, 64.2 years in group 3, and 63.1 years in group 4. Table 1 shows age and gender distribution among four groups. The male-to-female ratio in the control group was 1.1 to 1. In the study groups, it ranged from 1 to 1 to 1.2 to 1, with an average of 1.1 to 1.

**Table 1:** Age and gender distribution for patients in all groups

Group	Age (years)	Gender (male-to-female ratio)
Group 0	64.9	1.1:1
Group 1	62.4	1.2:1
Group 2	66.1	1.1:1
Group 3	64.2	1:1
Group 4	63.1	1.1:1

The distribution of patients in the study groups is shown in Table 2. Patients with diabetic retinopathy who did not receive intravitreal bevacizumab injection (group 1) included 244 patients (18.7%). Those who received one injection (group 2) were 306 patients (23.4%), and those who received two injections (group 3) were 352 patients (27%). The last group (group 4) included 404 patients (30.9%) who received three injections or more. The majority of patients in group 4 received only three injections in one year before their surgery (385 patients representing 95.3% of them), 17 patients (4.2%) received four injections, and two patients (0.5%) received five injections.

**Table 2:** Total number of patients and number of patients with posterior capsular rupture in all groups PCR: polymerase chain reaction

Group	Total number of patients	Number of patients with PCR	Percentage	p-value
Group 0 (control)	1440	20	1.4%	-
Group 1	244	4	1.6%	p>0.2
Group 2	306	6	2%	p>0.2
Group 3	352	8	2.3%	0.1
Group 4	404	21	5.2%	p<0.01
Total (study groups)	1306	39	3%	0.05

As shown in Table 2 above, posterior capsular rupture occurred more significantly in patients with group 4. It occurred in 21 patients, representing 5.2% of them, and was statistically significant (p<0.01). Among patients in group 4, 17 patients (4.2%) received four injections, and capsular rupture occurred in one of them, representing 5.9%, and two patients (0.5%) received five injections where one of them had capsular rupture.

In group 1, four patients (1.6%) had posterior capsular rupture, and in group 2, the posterior capsular rupture occurred in six patients (2%) while it occurred in eight patients (2.3%) of group 3. In the control group, the number of patients with posterior capsular rupture was 20 (1.4%).

Two hundred and forty-four patients (18.7%) had diabetic retinopathy but did not receive intravitreal bevacizumab injection (group 1), of which only four patients had posterior capsular rupture during surgery, representing 1.6% (Table 2). Rupture of the posterior capsule occurred more frequently as the number of injections increased. In patients who received only one injection, six patients had rupture of the posterior capsule among 306 patients (2%). The figure increased to 2.3% in eight patients with two injections and showed a greater increase in patients with three or more injections occurring in 21 patients (5.2%). In the control group, 20 patients (1.4%) had posterior capsular rupture during surgery.

## Discussion

Diabetic retinopathy is a major cause of blindness worldwide. It has many implications for the eye, and among them are cataracts and the development of retinopathy. Our institution, King Hussein Medical Center of the Royal Medical Services, is a tertiary referral center where we have daily specialized medical and surgical retina clinics that deal with patients with diabetic retinopathy. Previously, laser treatment was the mainstay of treatment for patients with diabetic retinopathy when advanced to a severe non-proliferative or proliferative stage or when macular edema needs treatment. Nowadays, intravitreal injections are widely used worldwide to treat diabetic retinopathy [10]. In our institution, we use different intravitreal injections such as bevacizumab, ranibizumab, and aflibercept. The most commonly used agent is bevacizumab (Avastin); therefore, we investigated its use in our study. Intravitreal bevacizumab is used off-label to treat several eye conditions, such as choroidal neovascularization in age-related macular degeneration, macular edema due to diabetes, or central retinal vein occlusion.

In addition to the occurrence of diabetic retinopathy, diabetic patients also develop cataracts and eventually will need phacoemulsification surgery. Posterior capsular rupture during surgery may cause many complications, such as vitreous prolapse, secondary glaucoma, retinal detachment, cystoid macular edema, and endophthalmitis.

Intravitreal bevacizumab injection has been implicated as a potential risk factor for posterior capsular rupture in cataract surgery. Capsular rupture may have devastating complications, rendering the patient aphakic or resulting in long-standing complications such as glaucoma and corneal decompensation. There are studies in the literature that highlight this issue. Some researchers suggested that multiple injections, 10 or more, are a risk for posterior capsular rupture [7], and others suggested even one injection may increase the risk [10-15]. It is agreed that increasing the number of injections will increase the risk of capsular rupture. In our study, we excluded patients with ocular factors that may increase the risk of capsular rupture other than intravitreal injection, such as poor pupil dilatation, pseudoexfoliation, previous ocular trauma, uveitis, and glaucoma. There was no gender or age difference between the study groups and the control group, as shown in Table 1. All surgeries were done by senior ophthalmic surgeons.

Twenty patients in the control group (1.4%) had posterior capsular rupture during surgery. When compared with the incidence of rupture in all study groups, the figure was higher (3% in 39 patients) but was not statistically significant (Table 2, 0.05<p<0.1). Comparing the figures in each study group alone with the control group, there was an increase in the incidence of posterior capsule rupture as the number of injections increased. The figures were not statistically significant in groups 2 and 3. The risk was 2% in six patients with one injection (p>0.2), and 2.3% in eight patients with two injections (0.1<p<0.2). On the other hand, the results were statistically significant in group 4 patients who received three or more injections (21 patients representing 5.2%, p<0.01). The majority of patients in group 4 received only three injections in one year before their surgery (385 patients representing 95.3% of them), 17 patients (4.2%) received four injections, and two patients (0.5%) received five injections. For the patients who received four injections, posterior capsular rupture occurred in one of them, representing 5.9%, and this result did not differ significantly from those who received three injections. In addition, two patients received five injections, and one of them had a capsular rupture, representing half of this category. Taking into consideration the sample of this subgroup (only two patients), we did not draw a conclusion about this result in our study.

For diabetic patients without previous intravitreal injection (group 1), four among 244 patients (1.6%) developed posterior capsular rupture; the risk was higher than in non-diabetic patients of the control group but not statistically significant (p>0.2). Analyzing the results we obtained for posterior capsular rupture in study groups, we noticed a dose-response relationship starting with 1.6% risk in patients who received one injection to 2.3% in patients who received two injections, with a jump that was statistically significant to 5.2% in patients who received three or more injections.

The results we found in our study emphasize taking extra precautions and planning before doing phacoemulsification surgery in patients who received multiple intravitreal injections one year prior to the surgery. Precautions include prompt assessment of patients before surgery looking for deepening of the anterior chamber and examining the anterior vitreous phase looking for condensation or signs of inflammation. Both deepening of the anterior chamber and anterior vitreous phase condensation may be seen if the posterior capsule was previously punctured. Also assessing lens stability, looking for phacodonesis, and assessing adequate pupillary dilatation are advised. Intraoperatively, we advise adapting the soft-shell technique using both dispersive and cohesive ophthalmic viscosurgical devices to maintain the anterior chamber and protect the corneal endothelium. In cases of poor pupil dilatation, capsular tension rings or iris hooks are advised.

The mechanism of increased risk of posterior capsular rupture during phacoemulsification in patients who received previous intravitreal injections is postulated to be multifactorial, though the exact mechanism is not investigated in the literature. A possible explanation is a direct puncture of the posterior capsule during the injection as we inject bevacizumab 4 mm posterior to the limbus in phakic eyes [16-17]. Another theory is biochemical changes in the capsule itself due to the proximity of injection to the posterior capsule [18-19]. In addition, diabetes itself may render the capsule weak and increase the risk of capsular rupture. We recommend conducting future research, including prospective studies referencing current gaps in the literature and investigating the mechanism of causing capsular rupture, such as the biochemical impact of bevacizumab on the lens capsule itself.

The large sample size in our study (1440 patients in the study group and 1306 in the control group) contributes to the strength, statistical significance, power, and reliability of the results. In addition, conducting the study in a tertiary referral center makes the findings resemble real-world clinical settings. On the contrary, there are some limitations that we tried to minimize in our study. The key limitation of the study is that it is retrospective in nature. We are planning to conduct a similar prospective study in our institution with similar settings to confirm and investigate the conclusions we found. Another limitation is whether the posterior capsular rupture was surgeon-related. We minimized this by including the surgeries done by a consultant senior ophthalmologist in our institution. Another limitation is that the cause of posterior capsular rupture may still be related to ocular factors rather than due to previous injection, though we excluded patients with ocular risk factors that may increase the risk of capsular rupture, such as pseudoexfoliation, previous trauma, uveitis, and small pupils. The duration of diabetes mellitus itself may be a cause for the risk of posterior capsular rupture, and this may be considered another limitation. We minimized this by including only patients with a history of diabetes of 10 years or more to standardize data in all groups.

## Conclusions

Intravitreal bevacizumab injection in diabetic patients may increase the risk for posterior capsular rupture in phacoemulsification cataract surgery, especially in patients who received three or more injections within one year prior to their surgery. As the number of injections increases, the risk will increase. Extra precaution and planning before surgery are advised before and during surgery in such patients, such as prompt assessment of patients before surgery looking for deepening of the anterior chamber and examining the anterior vitreous phase looking for condensation or signs of inflammation, assessing lens stability, and adequate pupillary dilatation. Intraoperatively, soft-shell technique and the use of capsular tension rings or iris hooks in patients with poor pupil dilatation are advised.

The mechanism of increased risk of posterior capsular rupture during phacoemulsification in patients who received previous intravitreal injections needs further investigation, whether it is due to direct puncture of the posterior capsule during the injection, biochemical changes in the capsule itself, or due to weakness to the capsule itself in diabetic patients. Future research, including prospective studies referencing current gaps in the literature and investigating the mechanism of causing capsular rupture, such as the biochemical impact of bevacizumab on the lens capsule itself, is recommended.
